# Remarkable Response to Cisplatin Doublet Chemotherapy in Pulmonary Metastasis With Left Atrial Extension From a Nasopharyngeal Cancer

**DOI:** 10.7759/cureus.13793

**Published:** 2021-03-09

**Authors:** Frank S Fan, Chen-Feng Chiu, Hsuan-hua Huang, Hwei-Fan Wendy Shu

**Affiliations:** 1 Section of Hematology and Oncology, Ministry of Health and Welfare Taitung Hospital, Taitung County, TWN; 2 Section of Pulmonology, Department of Internal Medicine, Ministry of Health and Welfare Feng Yuan Hospital, Taichung City, TWN; 3 Pathology, Ministry of Health and Welfare Feng Yuan Hospital, Taichung City, TWN

**Keywords:** nasopharyngeal cancer, pulmonary metastasis, left atrium invasion, epstein-barr virus, chemotherapy, paclitaxel, cisplatin, gemcitabine

## Abstract

A 60-year-old male patient whose nasopharyngeal carcinoma was brought to complete remission with induction chemotherapy composing of cisplatin plus fluorouracil and subsequent radiotherapy with intent of cure eight years ago presented with dyspnea due to left side massive pleural effusion with pleural seedings, left lower lobe huge space occupying lesion, and left atrial tumor extending from the intrapulmonary lesion through left inferior pulmonary veins. Pleural biopsy revealed a picture of nonkeratinizing squamous cell carcinoma positive for Epstein-Barr virus-encoded small RNAs in situ hybridization, leading to a diagnosis of late pulmonary metastases from the antecedent nasopharyngeal carcinoma. Systemic chemotherapy with initial cisplatin plus paclitaxel and subsequent cisplatin plus gemcitabine brought remarkable resolution to the malignant cardiac and intrathoracic lesions. So far as we know, this is the first case report of left atrial invasion from pulmonary metastasis of a nasopharyngeal carcinoma origin in the English literature.

## Introduction

Tumor invasion into pulmonary veins or left atrium from lung cancer is a not-so-rare phenomenon. The incidence was found to be 9.3% as evaluated with CT scan [1]. Despite multidisciplinary therapeutic approach, mainly surgical resection, the prognosis of such advanced disease status was poor with a five-year survival of 14% only [2]. Interestingly, left atrial extension from pulmonary metastasis of other primary sites has also been reported in a variety of occasional cases including Ewing sarcoma, renal cell carcinoma, colon cancer, rectal cancer, chondrosarcoma, and cervix cancer [3-8]. Herein, we present an extraordinary case of nasopharyngeal carcinoma which, initially brought to complete response by induction chemotherapy and local radiotherapy, recurred eight years later as a pulmonary metastasis with invasion into left atrium through left pulmonary veins. To the best of our knowledge, this is the first report of left atrial involvement by nasopharyngeal carcinoma in the English literature.

## Case presentation

A 60-year-old single male patient went to a regional teaching hospital in central Taiwan for help in May 2020. His chief complaints were progressive exertional dyspnea and shortness of breath for one month. Poor appetite and body weight loss were also noted. He had a cigarette smoking history of more than 30-package-year. Laboratory examination revealed normal white cell count but mild microcytic anemia with hemoglobin 10 g/dL, mean corpuscular volume 73.3 fl, probably due to an alpha-thalassemia trait, and moderate thrombocytosis with platelet count 630,000/mL, most likely reactive to the underlying malignancy. There was no iron deficiency. His blood sugar, liver, and renal functions were within normal reference ranges. Serum tumor markers including carcinoembryogenic antigen, cancer antigen 19.9, and squamous cell carcinoma antigen were not elevated.

**Figure 1:**
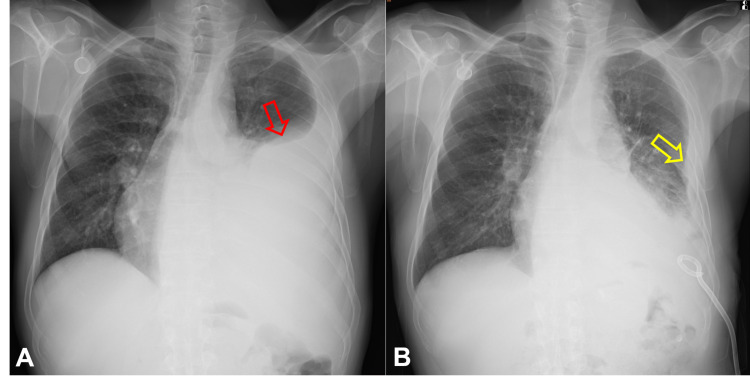
Chest X-ray films of the patient at presentation. A. Left side massive pleural effusion (red arrow) (May 6, 2020). B. Pleural thickening clearly seen (yellow arrow) after pleural effusion drainage with a pigtail catheter (May 18, 2020).

**Figure 2:**
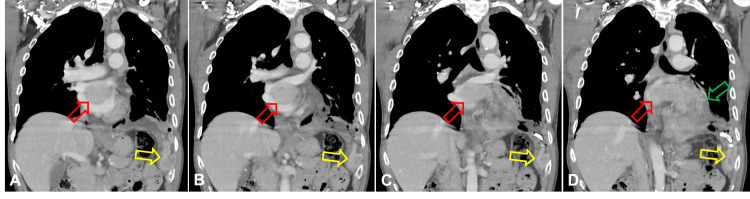
CT scan of chest at initial presentation (May 11, 2020). A-D: Subsequent coronal views. Red arrow: Tumor invasion into the left atrium from left inferior pulmonary veins. Yellow arrow: Pleural metastasis. Green arrow: Metastatic tumor mass occupying left lower lobe and pleura.

Chest X-ray examination revealed massive left side pleural effusion pushing heart shadow rightward (Figure 1A). For relieving respiratory distress, thoracocentesis with insertion of a draining catheter into the left pleural cavity (Figure 1B) and a left pleural biopsy were performed along with pleural effusion cytology studies. CT scan showed a huge mass, 9.3 cm in diameter, in the left lower lobe of the lung with tumor extension into left atrium through left inferior pulmonary veins (Figure 2). There were also obvious thickened pleural seedings.

**Figure 3:**
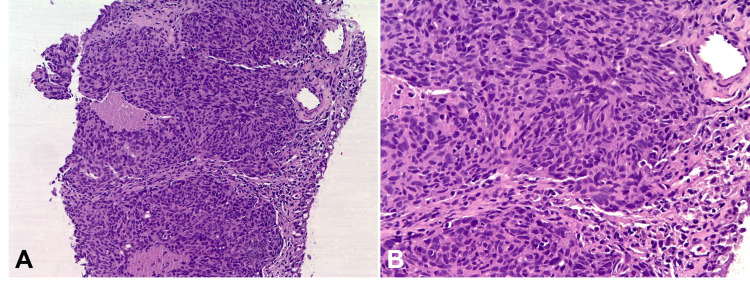
Pathology study of the pleural biopsy. Metastatic nonkeratinizing squamous cell carcinoma (hematoxylin and eosin stain; A. x100. B. x200).

The biopsy result turned out to be a nonkeratinizing squamous cell carcinoma (Figure 3). Since the patient had a history of nasopharyngeal carcinoma which was brought to complete remission after induction chemotherapy with one cycle of cisplatin plus continuous fluorouracil and subsequent radiotherapy at a tertiary hospital in 2012, in situ hybridization for Epstein-Barr virus-encoded small RNAs (EBER) was ordered for the present pleural biopsy and the first-hand nasopharyngeal pathologic specimen retrieved from the previous hospital. A CT scan of neck did not detect evidence of recurrence in nasopharynx or any regional lymph node metastasis.

**Figure 4:**
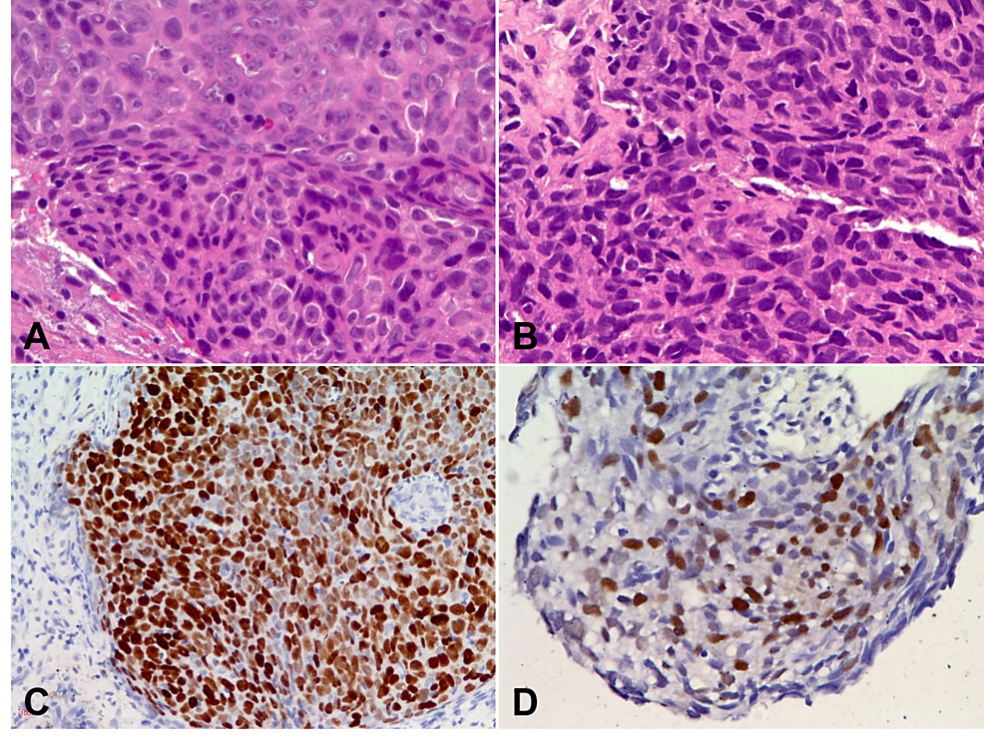
Pathology study of the primary and the late metastatic lesions. A and C. The original nasopharyngeal carcinoma. B and D. The present pleural metastasis. A and B: Hematoxylin and eosin stain (x400). C and D: EBER in situ hybridization (x400). EBER, Epstein-Barr virus-encoded small RNA

Because EBER study of both the present pleural and the original nasopharyngeal biopsies gave positive results and the microscopic pictures disclosed somewhat similarity in morphological characteristics (Figure 4), a diagnosis of pulmonary metastasis from an antecedent nasopharyngeal carcinoma is thus established. The squamous cell carcinoma marker p40 was also positive in samples taken from the earlier nasopharynx and the present pleura, respectively, with a time interval of eight years in between.

**Figure 5:**
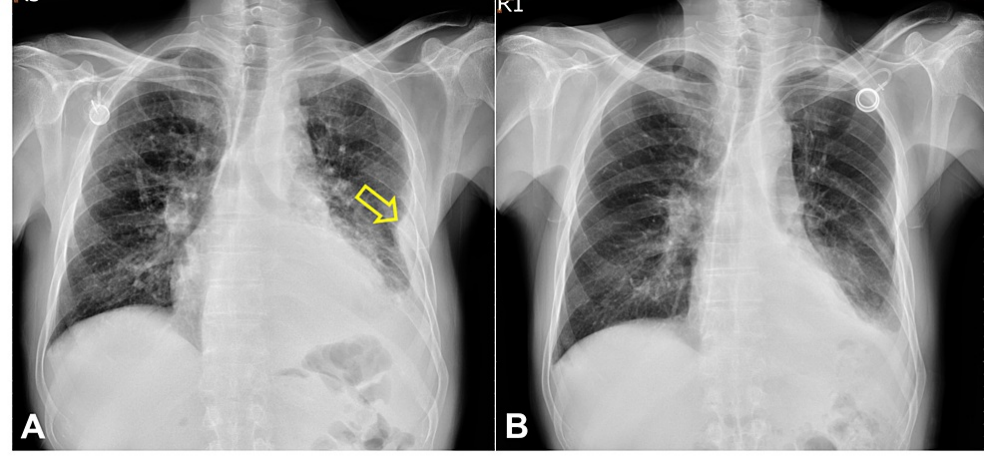
Chest X-ray films of the patient at the initial stage of treatment . A. After removal of the pigtail drainage catheter five days prior to the first course of cisplatin/paclitaxel chemotherapy (Jun. 8, 2020). Yellow arrow: pleural tumor seeding. B. Reimplantation of a Port-A catheter due to malfunction of the old one 25 days after the first course of cisplatin/paclitaxel chemotherapy (Jul. 8, 2020). Very good response in pleural seeding and effusion could be seen.

**Figure 6:**
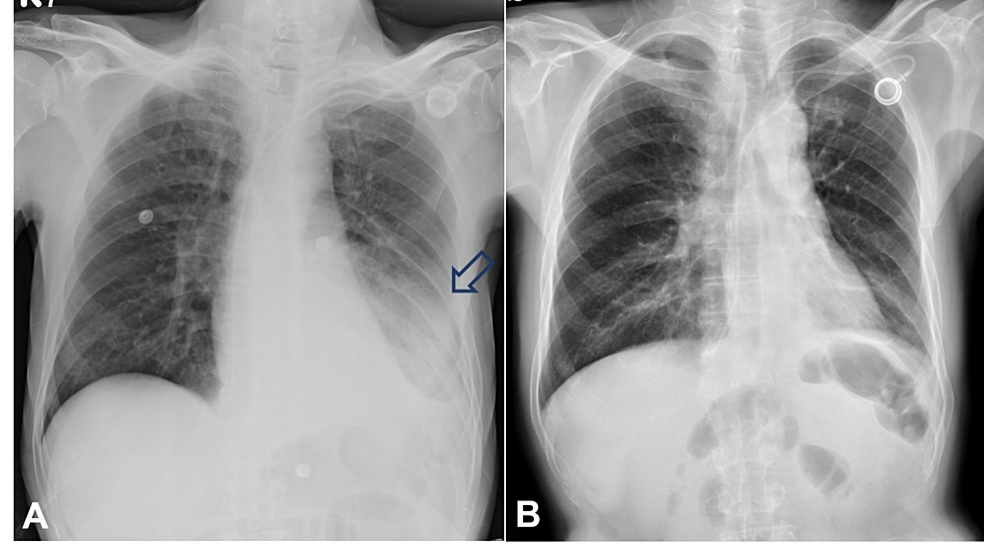
Chest X-ray films of the patient during treatment. A. Pneumonia in the left lower lobe (blue arrow) four days after the second cisplatin/paclitaxel chemotherapy (Jul. 18, 2020). B. Nearly complete resolution of the pulmonary metastasis and pneumonia eight days prior to the fourth course of cisplatin/gemcitabine chemotherapy (Jan. 6, 2021).

**Figure 7:**
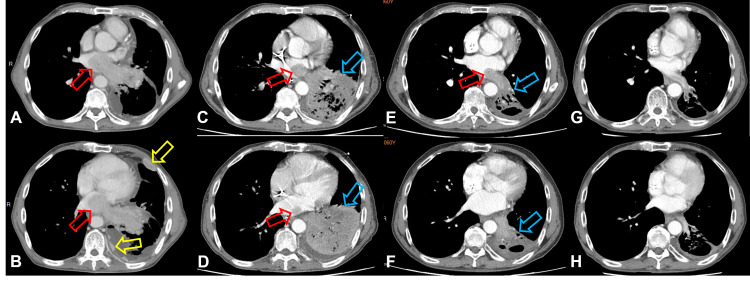
CT scan of chest during the whole treatment course. A and B. At initial presentation (May 11, 2020). C and D. Follow-up study after two courses of cisplatin/paclitaxel chemotherapy (Jul. 22, 2020). E and F. Follow-up study after four courses of cisplatin/paclitaxel chemotherapy (Oct. 7, 2020). G and H. Follow-up study after four additional courses of cisplatin/gemcitabine chemotherapy (Feb. 3, 2021). Red arrow: Tumor invasion into the left atrium from left inferior pulmonary veins. Yellow arrow: Pleural metastasis. Blue arrow: Left lower lobe metastatic tumor mixed with pneumonia consolidation.

The patient took our advice and agreed for a systemic combination chemotherapy with cisplatin (100 mg/m2) and paclitaxel (175 mg/m2). Surprisingly, the pulmonary lesions responded dramatically to the first course of chemotherapy (Figure 5). He was subsequently scheduled to take the chemotherapy every four weeks as the first step of therapy. Although the treatment plan was interrupted for a short period of time due to left side pneumonia with impending septic shock after the second course (Figure 6A), he eventually recovered (Figure 6B) and completed totally four courses of cisplatin/paclitaxel chemotherapy from June to September 2020. Although follow-up study with CT scan revealed a very good response with dramatic shrinkage of tumors in the pleura, lung, and heart, complete resolution of the lesions was not achieved (Figure 7A-F). He then received four more courses of chemotherapy composing of cisplatin (100 mg/m2) on day 1 plus gemcitabine (1000 mg/m2) on day 1 and 8 from October 2020 to January 2021. Interestingly, this second step of therapy brought nearly complete remission of the metastatic pulmonary lesions and left atrial tumor invasion (Figure 7G,H).

## Discussion

Traditionally, nasopharyngeal cancer is closely linked to Epstein-Barr virus infection [9] and the only subtype of lung cancer strongly associated with Epstein-Barr virus is lymphoepithelioma-like carcinoma of the lung in Asian patients [10], while our patient’s pleural lesions did not belong to this specific lung cancer category. Despite detection of Epstein-Barr virus genome or transcripts in a very small number of lung cancer tissues was reported recently, the incidence, 7 out of 1127, was quite low [11]. We thus tend to believe that late pulmonary and pleural metastases from a preceding nasopharyngeal carcinoma would be a more coherent diagnosis based on the patient’s past history and the rarity of Epstein-Barr virus in common lung cancer types.

The incidence of pulmonary metastasis from nasopharyngeal carcinoma is estimated to be 6.8% [12], and lung metastasis alone has a more favorable prognosis than metastasis to multiple or other sole organs [13]. Nonetheless, an awesome pulmonary metastasis with left atrial extension like our patient’s surely would not be considered to have an optimistic prognosis. Fortunately, the lesions responded to cisplatin-based systemic chemotherapy rather well and the risky cardiopulmonary distress was satisfactorily relieved.

Treatment with cisplatin plus paclitaxel as the first-line regimen for metastatic nasopharyngeal carcinoma led to comparable progression-free survival and overall survival to those of other four commonly adopted protocols including cisplatin + fluorouracil, cisplatin + gemcitabine, cisplatin + paclitaxel + fluorouracil, and cisplatin + fluorouracil + bleomycin according to a retrospective analysis [14]. Therefore, for nasopharyngeal carcinoma patients who had already received cisplatin plus fluorouracil as the front-line therapy, we like to recommend cisplatin in combination with either paclitaxel or gemcitabine as the salvage treatment upon metastatic relapse.

Modern induction chemotherapy has been shown to bring left atrial extension from lung cancer into remission successfully and make complete resection of residual pulmonary tumor possible without the aid of cardiopulmonary bypass, reducing the surgical risk to a great degree [15]. It seems confident to say that shrinkage of tumors invading left atrium with intensive systemic chemotherapy should also be the first step of treatment prior to surgical procedures of curative intent for chemotherapy-sensitive metastasis from other primary sites including nasopharyngeal carcinoma. Furthermore, in the era of molecular targeted therapy and immunotherapy, in addition to immune checkpoint inhibitors [16], rapid progress could be expected in the field of developing specific therapeutic agents targeting Epstein-Barr virus-related molecules [17-18], probably rendering invasive resection of even less priority in treating metastatic nasopharyngeal carcinoma a few years from now.

## Conclusions

A malignant tumor could relapse as a distant metastasis long after the primary site was cleared up. Modern pathologic investigation techniques provide tremendous help in identification of the tumor’s nature and aid in choosing the best therapeutic modality. Cancer invasion into left atrium along pulmonary veins might respond to adequate systemic therapy very well, making the patient exempt from high risk surgical excision. This case report gives an extremely encouraging demonstration.
